# Comparison of frequency of insulin resistance in patients with chronic obstructive pulmonary disease with normal controls

**DOI:** 10.12669/pjms.316.7983

**Published:** 2015

**Authors:** Zareen Kiran, Numan Majeed, Bader Faiyaz Zuberi

**Affiliations:** 1 Dr. Zareen Kiran, FCPS. Section of Endocrinology, Department of Medicine, Aga Khan University Hospital, Karachi, Pakistan; 2 Dr. Numan Majeed, MBBS. Department of Biochemistry, Ziauddin University, Karachi, Pakistan; 3 Dr. Bader Faiyaz Zuberi, FCPS. Department of Medicine, Dow University of Health Sciences, Karachi, Pakistan

**Keywords:** BMI, Chronic obstructive pulmonary disease, HOMA-IR, Insulin resistance

## Abstract

**Objective:**

To compare mean homeostatic model assessment of insulin resistance (HOMA-IR) in patients with and without chronic obstructive pulmonary disease (COPD).

**Methods:**

A Case control analytic study was conducted in medical outpatient department of Medial Unit-II of Dow University of Health Sciences from April 2013 to September 2013. All patients with the diagnosis of COPD were included as cases. Controls were age match healthy individuals with minor illnesses. Age, weight, height and forced expiratory volume in one second to forced vital capacity (FEV1/FVC) ratio were documented. Fasting blood glucose and fasting insulin levels were done. Body mass index (BMI) and IR was calculated using the formulas. HOMA-IR was compared between cases and controls.

**Results:**

Forty COPD patients were compared with thirty five age match controls. HOMA-IR was found to be higher in cases as compared to controls (2.85 v/s 2.00) with a p value <0.000.

**Conclusion:**

COPD is one of the chronic debilitating diseases in our region with various extra-pulmonary complications. We found IR to be present higher in COPD patients compared with healthy controls. Evaluating the pulmonary function as well as systemic metabolic parameters, may contribute to minimizing mortality and morbidity.

## INTRODUCTION

Chronic Obstructive Pulmonary Disease is a systemic disease characterized by the irreversible airflow obstruction. The airflow restriction is an abnormal inflammatory response occurring in the lungs against harmful particles and gases in the inhaled air.^1^ COPD is one of the leading causes of chronic morbidity and mortality worldwide^1^ the mortality due to this disease is increasing rapidly, making it third leading cause of death by 2020.^2^ COPD is preventable and treatable. Once considered primarily a pulmonary disease, it is now associated with a variety of systemic manifestations. It is characterized by low grade chronic inflammatory response in the body which is associated with several metabolic effects and systemic complications.^3^ One of the grave metabolic associations of any chronic inflammatory activity is insulin resistance.^4^,^5^

Insulin resistance is one of the leading metabolic and endocrine problems in the world. It is the mechanism behind and association in the manifestations of obesity^6^, dyslipidemias, cardiovascular^6^ and cerebrovascular morbidities and mortalities. In a recent study, it has been suggested to be associated with the progression of vascular endothelial dysfunction in patients with COPD.^7^ In one study, IR has been found to be higher in patients with COPD compared with healthy age-matched controls.^8^ It has been speculated that IR is a risk factor for the development of atherosclerotic cardiac disease and even type 2 diabetes mellitus in the future in patients with COPD.^8^ Moreover, it is independent of the severity of airway obstruction suggesting its early occurrence in the disease process.^3^ Thus, insulin resistance carries higher risk for metabolic and cardiovascular diseases in patients with COPD even when clinically stable.^3^

This study compares insulin resistance as mean HOMA-IR in patients with and without chronic obstructive pulmonary disease in the region of Pakistan. There is no study in our region about the association of IR in COPD patients, which is a public concern. Therefore, apart from assessing lung functions in patients with COPD, evaluating their associated conditions like insulin resistance will lead to better management and quality of life in these patients.

## METHODS

The study was conducted at Medial Unit-II of Dow University of Health Sciences from April 2013 to September 2013. All patients who were attending medical outpatient departmentwith the diagnosis of COPD for more than one year, according to the updated American Thoracic Society/European Respiratory Society (ATS/ERS) 9 and Global Initiative for Chronic Obstructive Lung Disease (GOLD) 1 guidelines were considered. Only those subjects were included in the study which fitted the following inclusion criteria: A post-bronchodilator forced expiratory volume in one second (FEV1)/forced vital capacity (FVC) < or equal to 0.7 confirming the presence of airflow limitation that is not fully reversible. They were further classified by the same criteria into four groups; mild (FEV1%pred ≥80), moderate (FEV1%pred 50-80), severe (FEV1%pred 30-50) and very severe (FEV1%pred <30). Patients with following conditions were excluded from the study: obstructive airways disease other than COPD (bronchiectasis, cystic fibrosis or fibrosis due to tuberculosis), smoking within the last one month, patients on steroids, known thyroid diseases, hypercortisolism, diabetes mellitus, hyperlipidemias, ischemic heart disease, non-pulmonary infective diseases, renal, cardiac or hepatic failures, autoimmune diseases and malignancies. Ethical approval was sought from ethics review committee of the institute (IRB-370/DUHS-13). A case control analysis was conducted in the total of forty patients who fitted in our criteria and were hence enrolled in the study after performing spirometry and taking informed consent. Thirty five age-matched healthy controls attending OPD with minor illnesses like gastroenteritis and viral flu were enrolled after taking informed consent.

Data was collected for age, gender, weight and height and FEV1/FVC ratio. Body Mass Index (BMI) was calculated and patients allocated to normal, under or overweight categories accordingly. Fasting blood samples were obtained by cubital venipuncture and then transferred to a single laboratory for analysis. Plasma fasting glucose level was measured enzymatically using an automated analyzer. Fasting plasma insulin were measured by radioimmunoassay. Insulin resistance was determined for all patients using HOMA-IR, where HOMA-IR = fasting glucose (mg/dl) x fasting insulin (mU/L)/405.^10^ The pulmonary function tests of all individuals were performed according to the criteria recommended by European Respiratory Society, using a computer-associated spirometer device (Vmax22D, Sensor Medics, California; USA). FVC, FEV1 and FEV1/FVC ratio were measured and absolute values and predicted percent of these parameters were evaluated. The best three tests were recorded.^11^

### Statistical Analysis

Data analysis was done using SPSS version 18.0. Descriptive statistics were calculated for all continuous variables. Mean +/- Standard Deviation (SD) was calculated for age, weight, height, BMI, fasting blood glucose levels and fasting Insulin levels. Frequencies & percentages for categorical variables were calculated. BMI and HOMA-IR with and without COPD between two groups were compared using T test. P-value ≤ 0.05 was taken as statistically significant.

## RESULTS

Forty cases of COPD (23 males and 17 females) and thirty five age match healthy controls (21 males and 14 females) were enrolled in the study. The age ranged from a minimum of 26 years and maximum of 60 years with a mean of 40.88 years ± 11.127 years. Body Mass Index was calculated as weight (in Kg) per height (in meters) squared. Mean BMI was calculated to be 24.96 Kg/m^2^±5.65in cases and 25.19 Kg/m^2^±5.54in controls with no difference between the two groups (p value=0.978). However, overall participants were more overweight in both groups [27 (67.50%) of the COPD patients and 24 (60%) of the controls]. The characteristics of the two study groups are shown in Table-I. Patients with COPD were divided into four groups by the severity of their disease according to the guidelines.^1^ There were no patients with mild or very severe severities. Thirty patients (75%) had moderate COPD and only ten (25%) were found to be severe. HOMA-IR was calculated for all patients and found to be higher among cases as compared to controls (Mean 2.85 v/s 2.00) with a significant difference noted statistically (60% v/s 14.28%, p value=0.000). Comparing each BMI category of total number of patients showed that BMI status does not affect the presence of insulin resistance as there are more overweight patients without insulin resistance (p value=0.038) as shown in Table-II. Statistical comparison among independent variables between two groups is shown in the Table-III. An important observation is that Fasting glucose levels have shown significant difference between the two groups (p value=0.011) which is an independent factor.

**Table-I:** Characteristics of the control group and COPD cases.

	Cases N=40	Controls N=35
Age (years)	40.88 (SD±11.12)	41.9 (SD±11.7)
Sex	Male=23, Female=17	Male=21, Female=14
BMI	24.96 Kg/m^2^ (SD± 5.65)	25.19 Kg/m^2^ (SD± 5.54)
Disease stage	Moderate COPD = 30 (75%) Severe = 10 (25%)	-

**Table-II:** Comparison of BMI groups with Insulin resistance.

BMI category	Insulin Resistance		P value
	Absent (n)	Present (n)	
Underweight	7	0	0.038
Normal	12	5	
Overweight	27	24	
Total	46	29	

**Table-III:** Statistical comparison among independent variables between two groups.

	Control	Cases (COPD)	
	Mean (SD)	Mean (SD)	P value
Fasting blood glucose (mg/dl)	89 (13.70)	98.13 (10.24)	0.011
Fasting insulin level	9.35 (2.92)	12.04 (4.75)	-
BMI	25.2 (5.55)	24.96 (5.65)	0.978
HOMA-IR	2.01(0.61)	2.86 (1.09)	0.000
FEV1/FVC	85.52 (5.53)	56.67 (10.48)	-

## DISCUSSION

Chronic Obstructive Pulmonary Disease is recently recognized to have clear extra-pulmonary effect.^12^,^13^ There is a well-known association of COPD with excess risk of cardiovascular disease and type 2 diabetes.^3^ Epidemiological data and several retrospective studies suggest that diabetes mellitus is much more common in patients with COPD than in healthy controls.^14^,^15^ Similarly, a recent observational study has also shown higher prevalence of metabolic syndrome in patients with COPD than in healthy subjects (57% v/s 40%).^16^ In fact, studies have shown that mortality in patients with COPD is mainly due to non-respiratory disorders such as cardiovascular diseases rather than the airway disease itself.^17^ Systemic inflammation has a clear association with the presence of metabolic syndrome in COPD patients.^17^ This systemic inflammation associated with COPD is an important concept behind various metabolic and cardiovascular outcomes in these patients.^3^,^18^

One of the major systemic effects of such a chronic inflammatory condition is insulin resistance.^5^ Insulin resistance is a growing pandemic all over the world. It is an important marker of metabolic syndrome in general, and is an independent risk factor for its cardiovascular complications. Systemic inflammation promotes IR, which in turn contributes to the development of metabolic syndrome in people with COPD.^3^ In developed regions, studies have also been carried out regarding various metabolic and inflammatory markers being present even in stable COPD patient population who have established metabolic syndrome.^13^ A recent observational multicenter study has measured various components of metabolic syndrome to be more frequent in COPD patients with prevalence of diabetes, osteoporosis, coronary artery disease and heart failure increasing their morbidity and mortality.^1^ Insulin resistance itself is also found to be present even in stable COPD patients because of the characteristic low grade inflammation [mean(SD), 3.71(3.63) vs. 1.83(1.17)].^8^

Comparative studies have demonstrated increased level of IR in COPD patients with metabolic syndrome than those without metabolic syndrome, reflecting prognostic significance in this subgroup with regards to their cardiovascular and metabolic outcomes.^19^,^20^ This finding however cannot be independently explained due to the chronic nature of the disease process and its underlying chronic inflammation, requiring more extensive research in this regard, especially in our population, because future therapies are now being considered to direct towards targeted inflammatory markers for appropriate management of this patient population.^18^ More recently, a cross sectional study from the National Health and Nutrition Evaluation Survey (NHANES) data set (2007–2010) has also identified many associated factors, including insulin resistance which has a direct contribution to the development of metabolic syndrome in patients with COPD.^21^ Naik et al explicitly explained that various metabolic factors are linked with COPD. These factors are contributing the pathogenic variety needing special attention.^22^ Therefore, as an essential part of the spectrum, still a direct effect of IR on the patients with COPD independent of other metabolic and inflammatory markers would remain an area of research as IR is a dynamic connection between a chronic inflammatory process like COPD and metabolic derangements.

However, no study has compared an independent association of IR in patients with COPD regardless of other metabolic markers with healthy controls. In a third world country like Pakistan, where COPD patient population is an important health care figure on the government and its policies, there is no study regarding its association with IR or any other metabolic parameters with its consequent effects on cardiovascular and endocrine outcomes. Moreover, studies are required to see the effect of managing IR as well as various other metabolic parameters in patients with COPD apart from their respiratory profiles to improve the morbidity and mortality.

Our study compares the presence of HOMA-IR in patients with COPD with healthy age matched controls. There was no difference in the BMI groups, with greater frequency of being overweight in both study populations. Therefore, insulin resistance was found to have an independent association with COPD patients regardless of other metabolic parameters. A recent retrospective observational analysis of such outpatient population of COPD has shown higher prevalence of diabetes mellitus with higher BMI and use of corticosteroids as an associated factor which might be contributing^15^, but our study has excluded such confounding factor. However, fasting blood sugar and fasting insulin levels were higher in the COPD patients as compared to healthy controls. This is an important independent finding which needs further research because this has directly affected the calculated insulin resistance which is higher among COPD patients (2.85 v/s 2.00, p value <0.000). Whether this reflects the finding of the general population is an area of controversial debate, as our sample size was small, which was a limitation to our study. The reason being the strict exclusion criteria to take control on confounders of insulin resistance and a justification of small size as well. Another important limitation is that our healthy control population was not ideal controls, which was due to our ethical consideration to population sampling.

## CONCLUSION

Insulin resistance is found to be higher in patients with COPD even when stable, compared with healthy controls in our tertiary setup. In the light of its well-known contribution to metabolic syndrome in this chronic disease and its progressive risk to cause type 2 diabetes and cardiovascular morbidity, it needs to be evaluated along with other markers in further studies. Moreover, it may be emphasized to evaluate COPD patients far beyond their respiratory condition, during their follow up as this may have impact on their health care outcomes.
